# Metabolic Influences That Regulate Dendritic Cell Function in Tumors

**DOI:** 10.3389/fimmu.2014.00024

**Published:** 2014-01-30

**Authors:** Han Dong, Timothy N. J. Bullock

**Affiliations:** ^1^Experimental Pathology Program, University of Virginia, Charlottesville, VA, USA; ^2^Department of Pathology, University of Virginia, Charlottesville, VA, USA

**Keywords:** dendritic cell, tumor-associated dendritic cell, activation, metabolism, glycolysis, oxidative phosphorylation, lipid metabolism, cancer immunotherapy

## Abstract

Dendritic cells (DC) are critical regulators of both activation and tolerance in the adaptive immune response. The dual nature of DC immunoregulatory function depends on their differentiation and activation status. DC found within the tumor microenvironment (TME) and tumor-draining lymph node often exist in an inactive state, which is thought to limit the adaptive immune response elicited by the growing tumor. The major determinants of DC activation and the functional alterations in DC that result from integrating exogenous stimuli have been well investigated. Extensive efforts have been made to elucidate how the TME contributes to the inactivated/dysfunctional phenotype of tumor-associated DC (TADC). Although performed predominantly on *in vitro* DC cultures, recent evidence indicates that DC undergo required, coordinated alterations in their metabolism upon activation, and dysregulated metabolism in TADC is associated with their reduced immunostimulatory capacity. In this review, we will focus on the role of glycolysis and fatty acid metabolism in DC activation and function and discuss how these metabolic pathways may be regulated in TADC. Further, we consider the need for developing novel experimental approaches to assess metabolic choices *in vivo*, and the necessity for integrating metabolic regulation into the optimized development of DC for tumor vaccines and immunotherapy for cancer.

## Introduction

**Figure 1:**
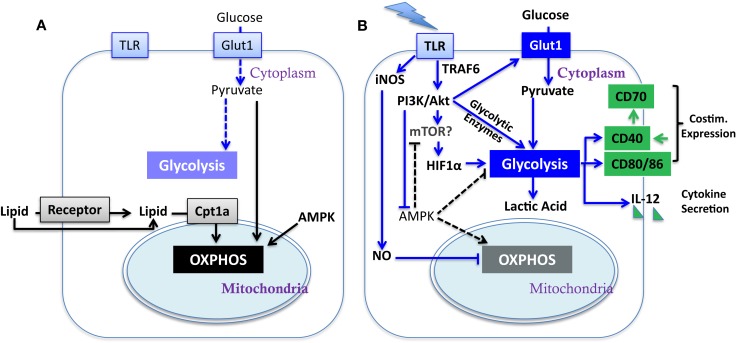
**Metabolic regulation of DC activation**. Illustrated here are metabolic pathways in resting DC **(A)** and activated DC **(B)**. Upon TLR stimulation, DC undergo a metabolic switch from OXPHOS (key mediators are linked by black arrows) to glycolysis (key mediators are linked by blue arrows), which contributes to their activation (major determinants in green; costim. is short for co-stimulatory molecule). Block arrows mark blockade and dash lines show insignificant process. The impact of mTOR on glycolysis has yet to be fully elucidated in DC (see Section “AKT and mTOR in DC Function” in text).

Dendritic cells (DC) serve as sentinels of the immune system. They constantly acquire antigen (Ag) from their environment and degrade it into short peptides that are presented at the cell surface in association with MHC molecules for surveillance by T cells. The inflammatory context in which DC exist influences their expression of critical co-stimulatory molecules and cytokines (Figure 1B) that provide the context for Ag presentation. Factors that promote the expression of co-stimulatory molecules and cytokines support the activation, expansion, and survival of responding T cells. In the absence of co-stimulation, DC present Ag in a manner that induces tolerance in the specific T cell repertoire, by mechanisms such as deletion (1) and anergy (2). During infection, inflammatory cytokines such as TNFα and type-1 interferons (IFN-1), or pathogen associated molecular patterns (PAMPs), induce a program of activation that initiates the CCR7-dependent migration of DC from the periphery to draining lymph nodes (3, 4). Additional stimulation via CD40 can further raise the activation state of DC, in part by inducing the expression of CD70 (Figure 1B) (5–7), leading to what is referred to as a licensed T cell response. While these basic tenets of DC activation have been well investigated, and extensively reviewed elsewhere (8, 9), recent studies have brought to light metabolic transitions in DC that are necessary for them to attain full function, or can regulate their functional activation. Here, we discuss the impact of these metabolic alterations on DC function; how metabolic pathways may be regulated in tumor-associated DC (TADC); and given the immature state of DC often found in tumors [and the negative prognosis associated with such immaturity (10, 11)] we consider the influence of the tumor microenvironment (TME) on these functions.

## Requirement for Glycolysis and DC Activation

Substantial evidence demonstrates that upon activation immune cells undergo a metabolic reprograming, switching from oxidative phosphorylation (OXPHOS) to aerobic glycolysis, a phenomenon initially observed in cancer cells in 1920s by Warburg (the Warburg effect) (12). In cancer cells, the Warburg effect is induced by growth factor signaling or by mutations in metabolism-related intrinsic pathways [such as loss-of-function mutants of succinate dehydrogenase (SDH) and Fumarate hydratase (FH), and constitutive activation of hypoxia-inducible factor-1 (HIF-1) and (c-Myc)] (13, 14), while T cells undergo the metabolic switch upon T cell receptor (TCR) activation by Ag in the context of proper co-stimulation (15). This change in cellular metabolic pathways provides essential metabolic and bio-energetic resources to support programs of new gene expression and protein synthesis during robust cellular proliferation. (16, 17)

A recent study from the Pearce group reported that PAMP stimulation of TLR induces a metabolic transition in resting immature DC, characterized by a conversion from mitochondrial β-oxidation of lipid and OXPHOS (Figure 1A) to aerobic glycolysis (Figure 1B) (18). Unlike in cancer cells and effector T cells, the Warburg effect in DC does not fuel cell division but rather appears to be crucial for DC activation and survival upon TLR stimulation. During the early phase (within 5 h) after exposure to TLR agonists, absence of glucose in culture medium led to profound defects in DC activation, including surface expression of CD40 and CD86 and production of IL-12p40 (Figure 1B). Subsequently, DC activated by TLR signals are highly reliant on glucose for survival and become more sensitive to death by nutrient limitation (18). Thus, initiating glycolysis at the time of DC activation is critical for full activation independent from its role in subsequent survival (18). The glycolytic pathway, rather than OXPHOS, may be required due to the need to generate substrates that will be used during DC activation. Alternatively, components of the glycolytic pathway, such as GAPDH, can directly regulate protein translation and may be responsible for regulating the translation of proteins that are critical for DC activation. Further studies will be necessary to elucidate the mechanism by which glycolytic pathway promotes the DC maturation process.

**Figure 2:**
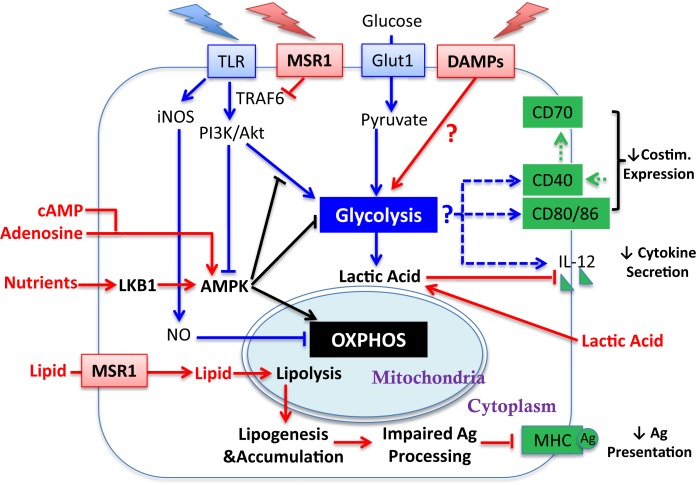
**Influence of tumor-derived factors on metabolic regulation of DC activation**. Illustrated here are tumor-derived factors (in red) and their impact/potential impact on metabolic pathways (linked by red arrows) for DC activation. Other symbols are defined the same way as in Figure 1. Lipid accumulation is detrimental to DC activation by impairing Ag processing. How the TME influences glycolytic switch and how that impacts DC activation requires further investigation (see Sections “Requirement for Glycolysis and DC Activation,” “Regulation of OXPHOS in DC,” and “AMPK Regulation of DC Function”).

The induction of glycolysis and DC maturation could be influenced by the tumor and the TME at several salient junctures (Figure 2). First, in the context of tumors, it is unclear whether the “find-me, eat-me” signals generated by damage associated molecular patterns (DAMPs)/alarmins, such as nucleotides, uric acid, heat shock proteins (HSP), HMBG1, and calreticulin [which stimulate DC in varied manners including via purinergic receptors (19), CD91 (20), TLR engagement (21, 22), RAGE (23), and TIM-3 (24)], are sufficient to promote glycolysis and DC maturation in the manner achieved with PAMP-mediated stimulation. Second, tumor-derived DC, or DC cultured with tumors, have been shown to be recalcitrant to TLR-mediated induction of CD40, CD86, and IL-12 (25), suggesting that the induction of glycolysis via this pathway in DC may be compromised. The mechanisms that regulate TLR function in mature DC after exposure to tumors have yet to be been elucidated, though inhibition of TLR signaling by MSR1 (see Section “MSR1 and DC Function” below) may contribute. Third, tumors are highly competitive for glucose; thus the substrate for glycolysis may be unavailable for DC and therefore the TME may not be permissive for the aspects of DC activation that are dependent upon glycolysis.

## AKT and mTOR in DC Function

TLRs activate PI3K in a MyD88-dependent manner (26). Similar to cancer cells (27, 28) and effector T cells (15, 29, 30), PI3K/AKT pathway has been implicated to play a pivotal role in controlling metabolic transition to glycolysis in TLR-stimulated DC (Figure 1B) (18). AKT promotes glycolysis in DC in part by increasing the expression of Glut-1 and likely activates mTOR. In T cells, AKT signaling promotes glycolysis by inducing the expression of rate-limiting enzymes such as hexokinase and phosphofructokinase (31) and activates mTOR. AKT is not the only driver of metabolic alterations in TLR-stimulated DC, as it is dispensable for the programed down-regulation of palmitate consumption after TLR stimulation. (18). Confounding data exist, however, about the contribution of mTOR (which is normally a downstream target of AKT) to DC immunostimulatory capacity as inhibition of mTOR by rapamycin in murine GM-CSF-driven DC and human myeloid DC prolongs their lifespan, promotes expression of co-stimulatory molecules and cytokines, and enhances DC immunogenicity (32, 33). Mouse DC treated with rapamycin were more effective at generating tumor immunity compared to untreated controls. (32). However, in contrast, rapamycin-treated monocyte-derived human DC expressed significantly lower levels of pro-inflammatory cytokines and had reduced capacity to elicit CD8^+^ T cell responses (33). Thus, while the TLR-induced activation of DC is dependent upon AKT-mediated induction of glycolysis, the contribution of mTORC1 [which is required to sustain glycolysis and effector functions in T cells (34)] to the glycolytic switch is unresolved, and sustained mTORC1 activation appears detrimental to the function of DC. Further studies are also needed to dissect the contribution of mTORC2 to any of these processes.

## Regulation of OXPHOS in DC

The underlying mechanisms for the AKT-independent reduction in OXPHOS upon TLR stimulation of DC have been recently studied using real-time metabolic flux analysis (35). The progressive impairment in OXPHOS in TLR-stimulated DC is due to inducible nitric oxide synthase (iNOS)-derived NO (Figure 1B). LPS stimulation induces *NOS2* mRNA and iNOS protein expression, and subsequent NO production in DC, presumably via IFN-1 or NF-κB-dependent mechanisms as reported in macrophages (36). The autocrine NO causes mitochondrial impairment and blocks OXPHOS, as reported previously in astrocytes (37) and macrophages (38). The mechanism of OXPHOS inhibition is likely by NO reversibly competing with oxygen to inhibit cytochrome *c* oxidase, the terminal enzyme of the electron transport chain (39). Thus, the increase in glycolysis in DC after TLR stimulation could be a survival response that serves to maintain cellular ATP levels and to prevent cell death when OXPHOS is blocked and pyruvate accumulates. Most interestingly, although switch to glycolysis has been demonstrated to be a direct consequence of iNOS-mediated OXPHOS blockade and is essential for the survival of iNOS-expressing DC *in vivo*, a long-term switch to glycolysis was shown to be dispensable for full DC activation. When NO production is inhibited, glycolysis is abrogated and β-oxidation is maintained in TLR-stimulated DC. Despite this, these DC showed unimpaired if not enhanced activation, as assessed by surface expression of MHC and co-stimulatory molecules, production of inflammatory cytokines, and capacity of DC to stimulate T cell proliferative responses at 24 h after TLR stimulation. Therefore, optimal DC function may require only a transient switch to glycolysis; this notion is consistent with the fact that either stimulation of CD40 (see below) or limiting mTOR activity promotes DC function, and raises an alternative hypothesis to explain DC dysfunction in tumors: that rather than being unresponsive to DAMPs, chronic exposure to DAMPS from tumors leads to a state of glycolysis-induced exhaustion or elimination of DC (Figure 2).

## AMPK Regulation of DC Function

The switch to glycolysis by DC is antagonized by adenosine monophosphate–activated protein kinase (AMPK), a master regulator of catabolic metabolism/OXPHOS in eukaryotic cells (Figure 1) (40, 41). AMPK can be induced by the nutrient sensor LKB1 (42), and functions in an opposing fashion with PI3K/AKT pathway to regulate TLR-induced metabolism and DC activation (Figure 2): up-regulation of AMPK in DC resulted in decreased LPS-induced IL-12p40 expression and glucose consumption, while suppression of AMPK by shRNAi leads to increased IL-12p40 and CD86 (18). Given the capacity of tumors to compete for glucose, one possible explanation for limited DC activity within the TME is the dominance of AMPK signaling over AKT-driven pathways (Figure 2). Intriguingly, IL-10, an inhibitor of DC activation, has been found to antagonize the TLR-induced hypophosphorylation of AMPK in TLR-stimulated DC, subsequently inhibiting the induction of glycolysis (18). AMPK activity is also strongly induced by cAMP (Figure 2). Along with adenosine, cAMP can skew myeloid cell differentiation to a tolerogenic DC format (43, 44) and ablate the function of already differentiated DC (45). It is therefore intriguing to consider whether the negative regulatory activity of cAMP and adenosine in tumor immunity is mediated by abrogating glycolysis. However, the increased immunostimulatory capacity of DC after mTOR blockade or iNOS inhibition is not simply due to a restoration of β-oxidation. If this were the case, then contrary to these observations, AMPK activation should promote DC immunostimulatory capacity. Further, ligation of CD40, which signals via TRAF-6, has a major influence on DC activation state and viability (46–49), and is a potent promoter of co-stimulatory molecule and IL-12 expression and immunity to cancer. TRAF-6 mediated signaling has been shown to promote fatty acid oxidation in CD8^+^ T cells (50) via activation of AMPK, raising the question as to how signals generated by CD40 engagement might be integrated into the metabolic programing initiated by TLR signaling. AMPK agonists have been proposed as anti-cancer agents due to their anti-Warburg effect in cancer cells (51), but this approach may be compromised by the detrimental effects of AMPK on DC and CD8^+^ T cell function. The role of AMPK in regulating glycolysis and β-oxidation in early and late stages of DC function requires further elucidation before it can be predicted how such an approach would impact on DC function in tumors.

## Lactic Acid Regulation of DC Function

Additional metabolic regulation of DC has been described. Endogenously produced lactic acid, the end product of glycolysis, accumulates in dense monocyte-derived DC cultures and tumor spheroids (52). Lactic acid concentration after glycolysis, rather than oxygen availability, skews DC differentiation into a tolerogenic orientation, as exemplified by increased production of IL-10 and loss of IL-12 (Figure 2) in response to TLR stimuli (53). This potentially identifies a negative feedback loop in DC function induced by glycolysis within activated DC, and may suggest that the beneficial effect of preventing the switch to glycolysis achieved by inhibiting NO production *in vitro* (35) could be a secondary consequence of avoiding lactic acid accumulation in DC culture. Interestingly, Ag uptake, MHC class I presentation and co-stimulatory molecule (CD40 and CD86) expression on DC can be increased by acidosis/extracellular acid (54), via acid-sensing ion channels (ASICs) (55). Thus, acidity and lactate accumulation may be independent variables on DC maturation. Although lactic acid buildup due to excessive DC density is unlikely to be a major consideration *in vivo*, tumor-generated lactic acid may serve this purpose (Figure 2) (27, 56, 57). Lactate export by cells is passive, mediated by monocarboxylate transporters (MCTs). A high extracellular lactate concentration in the TME could prevent its export from glycolytic DC, leading to lactate accumulation. It is also worth considering whether lactic acid buildup will differentially affect DC that are resident within tumors compared to those at the tumor edge, and whether lymph node resident DC are vulnerable to lactic acid prior to lymph node invasion by metastatic disease. Further investigations are necessary to establish a comprehensive understanding about how changes in glycolysis and OXPHOS influence activation and/or survival of different TADC subsets, whether DC maturation states are equivalently influenced by metabolic alterations, and whether diverse TLR and other PAMP stimuli have similar impact on DC metabolism.

## Lipid Uptake and Metabolism in TADC

While a switch to glycolytic metabolism is generally consistent with immune cell activation, fatty acid metabolism, and lipogenesis are thought to promote quiescence (17, 50). Several studies have now begun to illuminate a rather complex role of lipid, and lipid accumulation, in DC function, and how the presence or production of triglycerides (triacylglycerol, TAG) in the context of tumors may influence DC function.

As DC develop and mature, particularly after LPS stimulation, they take on a “lacy” appearance that is composed of an increased presence of fat and glycogen-containing lipid-body droplets (58). Notably, these high lipid DC (HL-DC) express higher levels of scavenger receptors including MARCO/MSR1, which may contribute to their accumulation of lipid (58). Aside from serving as a building block for many facets of DC biology, lipid can contribute to critical aspects of the ability of DC to perform their Ag processing and presentation functions. Cross-presentation of exogenous Ag on MHC class I molecules is highly dependent upon the presence of lipid bodies. Genetic inactivation of genes that regulate lipid-body assembly, or the use of diacylglycerol acyltransferase inhibitors that prevent TAG accumulation, abrogates the MHC class I cross-presenting capability of DC (59). Thus, lipid production and consumption play critical roles in DC biology.

## Lipid-Mediated Inhibition of TADC Function

It is therefore of interest that elevated levels of lipid, particularly TAG, were observed first by Herber and colleagues in DC during tumor progression of lymphoma, colon, and breast cancer in preclinical mouse models and cancer patients (60). The observed increased lipid accumulation is primarily a consequence of increased lipid uptake via up-regulated scavenger receptor A (SRA/MSR1/CD204) (Figure 2). Remarkably, considering the data from Bourgneres et al. (59), the major functional defect in HL-DC was a reduced capacity of DC to cross-present Ag (60). Normalization of lipid levels by a pharmacological inhibitor of acetyl-CoA carboxylase-1 (ACC-1), an enzyme that plays a critical role in lipogenesis, restored functional activity of lipid-laden DC, and enabled them to become more potent when used in a cancer vaccine (60). There are several notable aspects of this study that are worthy of further consideration. First, as mentioned above, lipid in DC by itself is not necessarily a marker of dysfunction. Indeed, a recent study examined the immunogenic qualities of liver-derived DC containing high and low amounts of lipid. HL-DC were considerably more immunogenic than their low lipid counterparts across multiple measurements (61). Further, Hwang and colleagues have demonstrated that saturated fatty acids can activate TLR4, leading to the up-regulation of MHC and costimulatory molecules. In contrast, polyunsaturated fats such as DHA, counteract the ability of saturated fats to induce DC maturation (62). Thus, rather than the amount of lipid within a DC being detrimental to function, the process by which lipid is acquired, or synthesized, or the type of lipid (saturated versus unsaturated) may be influential on DC function.

## MSR1 and DC Function

MSR1 has been shown to act both as a lipid receptor and as an innate pattern recognition receptor (PRR) that regulates inflammatory responses. As the first receptor identified for modified lipoproteins, the role of MSR1 has been well explored in pathogenesis of vascular disease particularly atherosclerosis (63). Besides modified self macromolecules, a wide range of PAMPs have been identified as MSR1 ligands, including bacterial surface components (e.g., LPS) and nucleic acids (e.g., CpG DNA and double-strand RNA), apoptotic cells, and endogenous danger molecules (64). Notably, the first report about negative effects of MSR1 in DC activation and function during adaptive immunity was presented by Yi and colleagues (65), in which they demonstrated that MSR1 suppresses the ability of TLR4 stimulation to license DC to prime naive CD8 T cells, drive their expansion, and promote their cytotoxic functionality both *in vitro* and *in vivo* (65). In agreement with data from Herber et al. they showed that lack of MSR1 in hematopoietic cells promoted tumor-protective immunity in a B16-OVA mouse melanoma model. In this model, MSR1 suppressed TLR4-induced activation of the transcription factor NF-κB by directly inhibiting ubiquitination of TRAF-6 (Figure 2) (66). However, the restriction of NF-κB activity by MSR1 can be independent of its ligand-binding domain, implying a novel signaling-regulatory role of MSR1 that can be uncoupled from its conventional role in endocytosis, including lipid uptake. Accordingly, one can speculate that up-regulation of MSR1 can contribute to DC dysfunction in cancer by skewing at least two pathways: (1) accumulation of lipids (2) suppression of TLR signaling. With respect to the second point, inhibition of TLR signaling may alter the balance between lipolysis and lipogenesis in favor of lipid accumulation. The therapeutic relevance of MSR1 and lipid uptake is reinforced by studies showing that direct targeting of MSR1 promotes tumor immunity (67, 68). Further, recent studies by Lerret et al. showed that the ability of total body irradiation (TBI), in combination with adoptive transfer of tumor-specific CD8^+^ T cells, to control established breast tumors may be achieved by promoting activation and function in tumor-resident DC via down-regulating MSR1 and inhibition of lipid uptake (69, 70). However, the tumor-derived factors that up-regulate MSR1 are poorly characterized, and it is yet to be definitively shown that lipid is an immunoregulatory ligand for MSR1on DC.

## MSR1-Independent Effects of Lipid on TADC

Although MSR1 engagement could account for poor DC function, additional influences of lipid on DC cannot be ruled out. Inhibition of ACC-1 resulted in normalization of lipid levels in TADC and was sufficient to restore functional activity in lipid-laden DC without changing expression of MHC and costimulatory molecules (60). This indicates that at least some accumulation of lipid in DC is due to *de novo* lipogenesis (Figure 2). Further, either the detrimental effects of lipid accumulation can be independent of MSR1 (as ACC-1 inhibition refunctionalizes TADC), or pathways released by ACC-1 inhibition can overcome MSR1-mediated inhibition. Evidence for the latter concept has been provide by Rehman et al. in a study demonstrating that ACC-1 inhibition enhances Ag capture (rather than Ag processing) by human DC (71). Confounding our understanding is that ACC-1 regulates the production of malonyl CoA, which in turn inhibits the activity of Carnitine palmitoyltransferase Ia (Cpt1a) (Figure 1A). Cpt1a strongly suppresses glycolysis via the Randle cycle, and knockdown of Cpt1a has been shown to strongly promote glycolysis in T cells (72). Thus, it is unclear why the inhibition of ACC-1, which should reduce glycolysis, enhances DC function unless (1) the lipogenesis program activates pathways that are significantly deleterious to DC function; (2) sustained glycolysis is indeed detrimental to DC function (discussed above); or (3) the presence of lipid is the detrimental factor, by influencing the availability of pyruvate for glycolysis rather than OXPHOS (73). Pointedly, it is uncertain why the accumulation of lipid might be detrimental to DC function at the level of Ag processing and presentation, especially given the importance of lipid bodies in this process. However, it has been shown that ceramides, which due to their hydrophobicity could accumulate in fat droplets, abrogate the ability of DC to uptake and present Ag (74) and also promote tumor-induced DC apoptosis (75).

## *In vitro* Veritas?

While the emerging picture of how alterations in DC metabolism can influence the function of DC, several words of caution should be written. One noticeable aspect of the majority of the studies cited in this review is that analysis of the contribution of metabolic alterations to DC function has generally been performed on DC generated from bone marrow or PBMC. This is necessitated by the rarity of DC in tissues, and the low sensitivity of the assays that are currently available to characterize metabolic activity. Thus, it is possible to posit that some metabolism-associated alterations described in these studies could be dependent upon the culture conditions that generate or sustain DC, and extrapolation to *in vivo* DC, particular to intratumoral DC, is not yet merited. DC, particular those of murine origin, generated via culture exist in a semi-activated functional state (our unpublished data) that may lead to different metabolic choices, and be influenced by different stimuli, compared to truly immature DC. This point is particularly salient when we consider some of the reported discrepancies on the impact of limiting or promoting glycolysis by modulating mTOR activity. Further, much work has yet to be done in defining whether metabolic alterations actually promote discrete functions of DC, or whether metabolic switching is a response to alterations in the nutrients in the immediate environment of the DC (tissue; lymphatics; lymph nodes, for example). However, the capacity for TLR to induce metabolic changes in DC in the consistent nutrient environment provided by *in vitro* culture, suggests that metabolic changes are not entirely due to alterations in the available nutrients, but rather these metabolic changes directly impact/regulate the activation and survival of DC. The single-cell analytical luxuries provided by flow cytometry have yet to be translated to metabolism studies, limiting our ability to make direct assessment of *in vivo* DC metabolic changes. Unfortunately, until radiotracer incorporation, extracellular flux assays, and mass spectrometry can be applied to 1000s of cells, rather than 100,000s, we will be dependent upon the use of fluorochrome-labeled substrates such as the glucose-derivative 2-NBDG to guide our impression of the metabolic pathways being used by DC derived from different *in vivo* environments.

## Summary

The metabolic and biochemical regulation of DC activation, function, and survival are just the beginning to be elucidated. Further understanding of this process will likely improve the quality and efficacy of DC expanded *ex vivo* for cancer vaccines [note the varied influences of cytokines on vaccine efficacy (9, 76)], as cytokines are known to impact metabolism. Further, metabolic re-invigoration of DC may provide an avenue for enhancing DC function in the TME or in tumor-draining lymph nodes, allowing for increased Ag processing and presentation after the induction of tumor damage, or in association with inhibition of checkpoint blockade molecules. Finally, approaches that promote the availability of glucose, or limit lipid uptake, in the TME might well increase the ability of TADC to activate and contribute to the adaptive immune responses elicited against tumors.

## Conflict of Interest Statement

The authors declare that the research was conducted in the absence of any commercial or financial relationships that could be construed as a potential conflict of interest.

## References

[1] Waithman J Allan RS Kosaka H Azukizawa H Shortman K Lutz MB Skin-derived dendritic cells can mediate deletional tolerance of class I-restricted self-reactive T cells. J Immunol (2007) 179:4535–41 17878350 10.4049/jimmunol.179.7.4535

[2] Appleman LJ Boussiotis VA T cell anergy and costimulation. Immunol Rev (2003) 192:161–80 10.1034/j.1600-065X.2003.00009.x 12670403

[3] Randolph GJ Angeli V Swartz MA Dendritic-cell trafficking to lymph nodes through lymphatic vessels. Nat Rev Immunol (2005) 5:617–28 10.1038/nri1670 16056255

[4] Alvarez D Vollmann EH von Andrian UH Mechanisms and consequences of dendritic cell migration. Immunity (2008) 29:325–42 10.1016/j.immuni.2008.08.006 18799141 PMC2818978

[5] Bullock TN Yagita H Induction of CD70 on dendritic cells through CD40 or TLR stimulation contributes to the development of CD8+ T cell responses in the absence of CD4+ T cells. J Immunol (2005) 174:710–7 15634890 10.4049/jimmunol.174.2.710

[6] Van Deusen KE Rajapakse R Bullock TN CD70 expression by dendritic cells plays a critical role in the immunogenicity of CD40-independent, CD4+ T cell-dependent, licensed CD8+ T cell responses. J Leukoc Biol (2010) 87:477–85 10.1189/jlb.0809535 19952354 PMC2830127

[7] Feau S Garcia Z Arens R Yagita H Borst J Schoenberger SP The CD4(+) T-cell help signal is transmitted from APC to CD8(+) T-cells via CD27-CD70 interactions. Nat Commun (2012) 3:948 10.1038/ncomms1948 22781761 PMC3606886

[8] Hammer GE Ma A Molecular control of steady-state dendritic cell maturation and immune homeostasis. Annu Rev Immunol (2013) 31:743–91 10.1146/annurev-immunol-020711-074929 23330953 PMC4091962

[9] Palucka K Banchereau J Dendritic-cell-based therapeutic cancer vaccines. Immunity (2013) 39:38–48 10.1016/j.immuni.2013.07.004 23890062 PMC3788678

[10] Treilleux I Blay JY Bendriss-Vermare N Ray-Coquard I Bachelot T Guastalla JP Dendritic cell infiltration and prognosis of early stage breast cancer. Clin Cancer Res (2004) 10:7466–74 10.1158/1078-0432.CCR-04-0684 15569976

[11] Perrot I Blanchard D Freymond N Isaac S Guibert B Pacheco Y Dendritic cells infiltrating human non-small cell lung cancer are blocked at immature stage. J Immunol (2007) 178:2763–9 17312119 10.4049/jimmunol.178.5.2763

[12] Warburg O Posener K Negelein E Ueber den Stoffwechsel der Tumoren. Biochem Zeitschr (1924) 152:319–44

[13] Frezza C Pollard PJ Gottlieb E Inborn and acquired metabolic defects in cancer. J Mol Med (Berl) (2011) 89:213–20 10.1007/s00109-011-0728-4 21301796 PMC3043233

[14] Porporato PE Dhup S Dadhich RK Copetti T Sonveaux P Anticancer targets in the glycolytic metabolism of tumors: a comprehensive review. Front Pharmacol (2011) 2:49 10.3389/fphar.2011.00049 21904528 PMC3161244

[15] Frauwirth KA Riley JL Harris MH Parry RV Rathmell JC Plas DR The CD28 signaling pathway regulates glucose metabolism. Immunity (2002) 16:769–77 10.1016/S1074-7613(02)00323-0 12121659

[16] Palsson-McDermott EM O’Neill LA The Warburg effect then and now: from cancer to inflammatory diseases. Bioessays (2013) 35:965–73 10.1002/bies.201300084 24115022

[17] Pearce EL Pearce EJ Metabolic pathways in immune cell activation and quiescence. Immunity (2013) 38:633–43 10.1016/j.immuni.2013.04.005 23601682 PMC3654249

[18] Krawczyk CM Holowka T Sun J Blagih J Amiel E DeBerardinis RJ Toll-like receptor-induced changes in glycolytic metabolism regulate dendritic cell activation. Blood (2010) 115:4742–9 10.1182/blood-2009-10-249540 20351312 PMC2890190

[19] Elliott MR Chekeni FB Trampont PC Lazarowski ER Kadl A Walk SF Nucleotides released by apoptotic cells act as a find-me signal to promote phagocytic clearance. Nature (2009) 461:282–6 10.1038/nature08296 19741708 PMC2851546

[20] Binder RJ Han DK Srivastava PK CD91: a receptor for heat shock protein gp96. Nat Immunol (2000) 1:151–5 10.1038/77835 11248808

[21] Vabulas RM Wagner H Schild H Heat shock proteins as ligands of toll-like receptors. Curr Top Microbiol Immunol (2002) 270:169–84 12467251 10.1007/978-3-642-59430-4_11

[22] Park JS Svetkauskaite D He Q Kim JY Strassheim D Ishizaka A Involvement of toll-like receptors 2 and 4 in cellular activation by high mobility group box 1 protein. J Biol Chem (2004) 279:7370–7 10.1074/jbc.M306793200 14660645

[23] Yang D Chen Q Yang H Tracey KJ Bustin M Oppenheim JJ High mobility group box-1 protein induces the migration and activation of human dendritic cells and acts as an alarmin. J Leukoc Biol (2007) 81:59–66 10.1189/jlb.0306180 16966386

[24] Chiba S Baghdadi M Akiba H Yoshiyama H Kinoshita I Dosaka-Akita H Tumor-infiltrating DCs suppress nucleic acid-mediated innate immune responses through interactions between the receptor TIM-3 and the alarmin HMGB1. Nat Immunol (2012) 13:832–42 10.1038/ni.2376 22842346 PMC3622453

[25] Idoyaga J Moreno J Bonifaz L Tumor cells prevent mouse dendritic cell maturation induced by TLR ligands. Cancer Immunol Immunother (2007) 56:1237–50 10.1007/s00262-006-0275-y 17237931 PMC11029892

[26] Gelman AE LaRosa DF Zhang J Walsh PT Choi Y Sunyer JO The adaptor molecule MyD88 activates PI-3 kinase signaling in CD4+ T cells and enables CpG oligodeoxynucleotide-mediated costimulation. Immunity (2006) 25:783–93 10.1016/j.immuni.2006.08.023 17055754 PMC2840381

[27] Levine AJ Puzio-Kuter AM The control of the metabolic switch in cancers by oncogenes and tumor suppressor genes. Science (2010) 330:1340–4 10.1126/science.1193494 21127244

[28] Ran C Liu H Hitoshi Y Israel MA Proliferation-independent control of tumor glycolysis by PDGFR-mediated AKT activation. Cancer Res (2013) 73:1831–43 10.1158/0008-5472.CAN-12-2460 23322009

[29] Engelman JA Luo J Cantley LC The evolution of phosphatidylinositol 3-kinases as regulators of growth and metabolism. Nat Rev Genet (2006) 7:606–19 10.1038/nrg1879 16847462

[30] Jones RG Thompson CB Revving the engine: signal transduction fuels T cell activation. Immunity (2007) 27:173–8 10.1016/j.immuni.2007.07.008 17723208

[31] Frauwirth KA Thompson CB Regulation of T lymphocyte metabolism. J Immunol (2004) 172:4661–5 15067038 10.4049/jimmunol.172.8.4661

[32] Amiel E Everts B Freitas TC King IL Curtis JD Pearce EL Inhibition of mechanistic target of rapamycin promotes dendritic cell activation and enhances therapeutic autologous vaccination in mice. J Immunol (2012) 189:2151–8 10.4049/jimmunol.1103741 22826320 PMC3424310

[33] Haidinger M Poglitsch M Geyeregger R Kasturi S Zeyda M Zlabinger GJ A versatile role of mammalian target of rapamycin in human dendritic cell function and differentiation. J Immunol (2010) 185:3919–31 10.4049/jimmunol.1000296 20805416

[34] Finlay DK Rosenzweig E Sinclair LV Feijoo-Carnero C Hukelmann JL Rolf J PDK1 regulation of mTOR and hypoxia-inducible factor 1 integrate metabolism and migration of CD8+ T cells. J Exp Med (2012) 209:2441–53 10.1084/jem.20112607 23183047 PMC3526360

[35] Everts B Amiel E van der Windt GJ Freitas TC Chott R Yarasheski KE Commitment to glycolysis sustains survival of NO-producing inflammatory dendritic cells. Blood (2012) 120:1422–31 10.1182/blood-2012-03-419747 22786879 PMC3423780

[36] Lewis RS Kolesnik TB Kuang Z D’Cruz AA Blewitt ME Masters SL TLR regulation of SPSB1 controls inducible nitric oxide synthase induction. J Immunol (2011) 187:3798–805 10.4049/jimmunol.1002993 21876038

[37] Bolanos JP Peuchen S Heales SJ Land JM Clark JB Nitric oxide-mediated inhibition of the mitochondrial respiratory chain in cultured astrocytes. J Neurochem (1994) 63:910–6 10.1046/j.1471-4159.1994.63030910.x 7519665

[38] Garedew A Moncada S Retraction. Mitochondrial dysfunction and HIF1alpha stabilization in inflammation. J Cell Sci (2012) 125:3254 10.1242/jcs.115949 18827009

[39] Cleeter MW Cooper JM Darley-Usmar VM Moncada S Schapira AH Reversible inhibition of cytochrome c oxidase, the terminal enzyme of the mitochondrial respiratory chain, by nitric oxide. Implications for neurodegenerative diseases. FEBS Lett (1994) 345:50–4 10.1016/0014-5793(94)00424-2 8194600

[40] Hardie DG Roles of the AMP-activated/SNF1 protein kinase family in the response to cellular stress. Biochem Soc Symp (1999) 64:13–27 10207618

[41] Hardie DG Ross FA Hawley SA AMPK: a nutrient and energy sensor that maintains energy homeostasis. Nat Rev Mol Cell Biol (2012) 13:251–62 10.1038/nrm3311 22436748 PMC5726489

[42] Tamas P Macintyre A Finlay D Clarke R Feijoo-Carnero C Ashworth A LKB1 is essential for the proliferation of T-cell progenitors and mature peripheral T cells. Eur J Immunol (2010) 40:242–53 10.1002/eji.200939677 19830737 PMC2988414

[43] Novitskiy SV Ryzhov S Zaynagetdinov R Goldstein AE Huang Y Tikhomirov OY Adenosine receptors in regulation of dendritic cell differentiation and function. Blood (2008) 112:1822–31 10.1182/blood-2008-02-136325 18559975 PMC2518889

[44] Challier J Bruniquel D Sewell AK Laugel B Adenosine and cAMP signalling skew human dendritic cell differentiation towards a tolerogenic phenotype with defective CD8(+) T-cell priming capacity. Immunology (2013) 138:402–10 10.1111/imm.12053 23278551 PMC3719950

[45] Panther E Durk T Ferrari D Di Virgilio F Grimm M Sorichter S AMP affects intracellular Ca2+ signaling, migration, cytokine secretion and T cell priming capacity of dendritic cells. PLoS One (2012) 7:e37560 10.1371/journal.pone.0037560 22624049 PMC3356328

[46] Miga AJ Masters SR Durell BG Gonzalez M Jenkins MK Maliszewski C Dendritic cell longevity and T cell persistence is controlled by CD154-CD40 interactions. Eur J Immunol (2001) 31:959–65 10.1002/1521-4141(200103)31:3<959::AID-IMMU959>3.0.CO;2-A 11241301

[47] Mackey MF Wang Z Eichelberg K Germain RN Distinct contributions of different CD40 TRAF binding sites to CD154-induced dendritic cell maturation and IL-12 secretion. Eur J Immunol (2003) 33:779–89 10.1002/eji.200323729 12616498

[48] Kobayashi T Walsh PT Walsh MC Speirs KM Chiffoleau E King CG TRAF6 is a critical factor for dendritic cell maturation and development. Immunity (2003) 19:353–63 10.1016/S1074-7613(03)00230-9 14499111

[49] Elgueta R Benson MJ de Vries VC Wasiuk A Guo Y Noelle RJ Molecular mechanism and function of CD40/CD40L engagement in the immune system. Immunol Rev (2009) 229:152–72 10.1111/j.1600-065X.2009.00782.x 19426221 PMC3826168

[50] Pearce EL Walsh MC Cejas PJ Harms GM Shen H Wang LS Enhancing CD8 T-cell memory by modulating fatty acid metabolism. Nature (2009) 460:103–7 10.1038/nature08097 19494812 PMC2803086

[51] Faubert B Boily G Izreig S Griss T Samborska B Dong Z AMPK is a negative regulator of the Warburg effect and suppresses tumor growth in vivo. Cell Metab (2013) 17:113–24 10.1016/j.cmet.2012.12.001 23274086 PMC3545102

[52] Gottfried E Kunz-Schughart LA Ebner S Mueller-Klieser W Hoves S Andreesen R Tumor-derived lactic acid modulates dendritic cell activation and antigen expression. Blood (2006) 107:2013–21 10.1182/blood-2005-05-1795 16278308

[53] Nasi A Fekete T Krishnamurthy A Snowden S Rajnavolgyi E Catrina AI Dendritic cell reprogramming by endogenously produced lactic acid. J Immunol (2013) 191:3090–9 10.4049/jimmunol.1300772 23956421

[54] Vermeulen M Giordano M Trevani AS Sedlik C Gamberale R Fernandez-Calotti P Acidosis improves uptake of antigens and MHC class I-restricted presentation by dendritic cells. J Immunol (2004) 172:3196–204 14978127 10.4049/jimmunol.172.5.3196

[55] Tong J Wu WN Kong X Wu PF Tian L Du W Acid-sensing ion channels contribute to the effect of acidosis on the function of dendritic cells. J Immunol (2011) 186:3686–92 10.4049/jimmunol.1001346 21321108

[56] Hirschhaeuser F Sattler UG Mueller-Klieser W Lactate: a metabolic key player in cancer. Cancer Res (2011) 71:6921–5 10.1158/0008-5472.CAN-11-1457 22084445

[57] Dhup S Dadhich RK Porporato PE Sonveaux P Multiple biological activities of lactic acid in cancer: influences on tumor growth, angiogenesis and metastasis. Curr Pharm Des (2012) 18:1319–30 10.2174/138161212799504902 22360558

[58] Maroof A English NR Bedford PA Gabrilovich DI Knight SC Developing dendritic cells become ‘lacy’ cells packed with fat and glycogen. Immunology (2005) 115:473–83 10.1111/j.1365-2567.2005.02181.x 16011516 PMC1782181

[59] Bougneres L Helft J Tiwari S Vargas P Chang BH Chan L A role for lipid bodies in the cross-presentation of phagocytosed antigens by MHC class I in dendritic cells. Immunity (2009) 31:232–44 10.1016/j.immuni.2009.06.022 19699172 PMC2803012

[60] Herber DL Cao W Nefedova Y Novitskiy SV Nagaraj S Tyurin VA Lipid accumulation and dendritic cell dysfunction in cancer. Nat Med (2010) 16:880–6 10.1038/nm.2172 20622859 PMC2917488

[61] Ibrahim J Nguyen AH Rehman A Ochi A Jamal M Graffeo CS Dendritic cell populations with different concentrations of lipid regulate tolerance and immunity in mouse and human liver. Gastroenterology (2012) 143:1061–72 10.1053/j.gastro.2012.06.003 22705178 PMC3459067

[62] Weatherill AR Lee JY Zhao L Lemay DG Youn HS Hwang DH Saturated and polyunsaturated fatty acids reciprocally modulate dendritic cell functions mediated through TLR4. J Immunol (2005) 174:5390–7 15843537 10.4049/jimmunol.174.9.5390

[63] Suzuki H Kurihara Y Takeya M Kamada N Kataoka M Jishage K A role for macrophage scavenger receptors in atherosclerosis and susceptibility to infection. Nature (1997) 386:292–6 10.1038/386292a0 9069289

[64] Areschoug T Gordon S Scavenger receptors: role in innate immunity and microbial pathogenesis. Cell Microbiol (2009) 11:1160–9 10.1111/j.1462-5822.2009.01326.x 19388903

[65] Yi H Yu X Gao P Wang Y Baek SH Chen X Pattern recognition scavenger receptor SRA/CD204 down-regulates Toll-like receptor 4 signaling-dependent CD8 T-cell activation. Blood (2009) 113:5819–28 10.1182/blood-2008-11-190033 19349620 PMC2700321

[66] Yu X Yi H Guo C Zuo D Wang Y Kim HL Pattern recognition scavenger receptor CD204 attenuates Toll-like receptor 4-induced NF-kappaB activation by directly inhibiting ubiquitination of tumor necrosis factor (TNF) receptor-associated factor 6. J Biol Chem (2011) 286:18795–806 10.1074/jbc.M111.224345 21460221 PMC3099696

[67] Yi H Guo C Yu X Gao P Qian J Zuo D Targeting the immunoregulator SRA/CD204 potentiates specific dendritic cell vaccine-induced T-cell response and antitumor immunity. Cancer Res (2011) 71:6611–20 10.1158/0008-5472.CAN-11-1801 21914786 PMC3213980

[68] Guo C Yi H Yu X Zuo D Qian J Yang G In situ vaccination with CD204 gene-silenced dendritic cell, not unmodified dendritic cell, enhances radiation therapy of prostate cancer. Mol Cancer Ther (2012) 11:2331–41 10.1158/1535-7163.MCT-12-0164 22896667 PMC3496075

[69] Lerret NM Rogozinska M Jaramillo A Marzo AL Adoptive transfer of mammaglobin-A epitope specific CD8 T cells combined with a single low dose of total body irradiation eradicates breast tumors. PLoS One (2012) 7:e41240 10.1371/journal.pone.0041240 22911764 PMC3401129

[70] Lerret NM Marzo AL Adoptive T-cell transfer combined with a single low dose of total body irradiation eradicates breast tumors. Oncoimmunology (2013) 2:e22731 10.4161/onci.22731 23525138 PMC3601156

[71] Rehman A Hemmert KC Ochi A Jamal M Henning JR Barilla R Role of fatty-acid synthesis in dendritic cell generation and function. J Immunol (2013) 190:4640–9 10.4049/jimmunol.1202312 23536633 PMC3633656

[72] van der Windt GJ Everts B Chang CH Curtis JD Freitas TC Amiel E Mitochondrial respiratory capacity is a critical regulator of CD8+ T cell memory development. Immunity (2012) 36:68–78 10.1016/j.immuni.2011.12.007 22206904 PMC3269311

[73] Sabbah HH Stanley WC Partial fatty acid oxidation inhibitors: a potentially new class of drugs for heart failure. Eur J Heart Fail (2002) 4:3–6 10.1016/S1388-9842(01)00183-0 11812659

[74] Sallusto F Nicolo C De Maria R Corinti S Testi R Ceramide inhibits antigen uptake and presentation by dendritic cells. J Exp Med (1996) 184:2411–6 10.1084/jem.184.6.2411 8976196 PMC2196395

[75] Kanto T Kalinski P Hunter OC Lotze MT Amoscato AA Ceramide mediates tumor-induced dendritic cell apoptosis. J Immunol (2001) 167:3773–84 11564794 10.4049/jimmunol.167.7.3773

[76] Palucka K Banchereau J Cancer immunotherapy via dendritic cells. Nat Rev Cancer (2012) 12:265–77 10.1038/nrc3258 22437871 PMC3433802

